# Genetic Basis of Growth Adaptation of *Escherichia coli* after Deletion of *pgi*, a Major Metabolic Gene

**DOI:** 10.1371/journal.pgen.1001186

**Published:** 2010-11-04

**Authors:** Pep Charusanti, Tom M. Conrad, Eric M. Knight, Karthik Venkataraman, Nicole L. Fong, Bin Xie, Yuan Gao, Bernhard Ø. Palsson

**Affiliations:** 1 Department of Bioengineering, University of California San Diego, La Jolla, California, United States of America; 2 Center for the Study of Biological Complexity, Virginia Commonwealth University, Richmond, Virginia, United States of America; 3 Department of Computer Science, Virginia Commonwealth University, Richmond, Virginia, United States of America; Universidad de Sevilla, Spain

## Abstract

Bacterial survival requires adaptation to different environmental perturbations such as exposure to antibiotics, changes in temperature or oxygen levels, DNA damage, and alternative nutrient sources. During adaptation, bacteria often develop beneficial mutations that confer increased fitness in the new environment. Adaptation to the loss of a major non-essential gene product that cripples growth, however, has not been studied at the whole-genome level. We investigated the ability of *Escherichia coli* K-12 MG1655 to overcome the loss of phosphoglucose isomerase (*pgi*) by adaptively evolving ten replicates of *E. coli* lacking *pgi* for 50 days in glucose M9 minimal medium and by characterizing endpoint clones through whole-genome re-sequencing and phenotype profiling. We found that 1) the growth rates for all ten endpoint clones increased approximately 3-fold over the 50-day period; 2) two to five mutations arose during adaptation, most frequently in the NADH/NADPH transhydrogenases *udhA* and *pntAB* and in the stress-associated sigma factor *rpoS*; and 3) despite similar growth rates, at least three distinct endpoint phenotypes developed as defined by different rates of acetate and formate secretion. These results demonstrate that *E. coli* can adapt to the loss of a major metabolic gene product with only a handful of mutations and that adaptation can result in multiple, alternative phenotypes.

## Author Summary

Bacteria must constantly adapt to many different environmental challenges, but how do they adapt when they lose a key gene product? We addressed this question using *Escherichia coli* lacking *pgi*, a major metabolic gene involved in sugar utilization, by serially passing replicates lacking the *pgi* gene for 50 days and resequencing the entire genome from endpoint clones isolated from nine of the replicates. We repeatedly found mutations in *rpoS*, a gene mostly active during stationary phase but one that regulates the expression of about fifty genes during exponential phase growth, and *udhA* and *pntAB*, three genes involved in maintaining redox balance in the cell. We also found multiple distinct endpoint phenotypes; the replicates could be stratified into three different groups after adaptation based on their rates of acetate and formate secretion. These results support the view that the metabolic network in *E. coli* is robust and can adjust to loss of a major metabolic gene in alternative ways.

## Introduction

Recent advances in DNA sequencing technology enable bacterial genomes to be fully sequenced with a resolution high enough to find all differences relative to a reference sequence. These developments make possible the study, at the whole-genome level, of the genetic basis through which bacteria adapt to different perturbations. For example, *E. coli* adaptively evolved to achieve optimal growth on glycerol were found to have two to three mutations when endpoint clones were re-sequenced and compared to the parental wild-type strain [1]. Allelic replacement introducing the discovered mutations into the parental strain was then used to show the phenotypic causality of each mutation [1]. In another example, a long-term adaptive evolution study of *E. coli* revealed that genomic evolution did not decrease over time as expected. Instead, genomic evolution remained nearly constant over 20,000 generations and nearly all of the mutations that appeared were beneficial [2], [3]. Other adaptive evolution and resequencing-based studies have examined at the genome level how *E. coli* adapts to growth on other carbon sources besides glycerol [4]–[6], how *Myxococcus xanthus* transitions from cooperative behavior to cheating and back [7], and how different pathogens develop antibiotic resistance [8]–[11].

Another topic that has received intense investigation is that of compensatory mutations, especially with regard to antibiotic resistance [12]. Bacteria that develop antibiotic resistance through mutation of the target enzyme or through horizontal gene transfer (HGT) are often less fit than their drug-sensitive counterparts, but wild-type fitness levels can sometimes be restored if they acquire additional mutations that compensate for the lower fitness. Crucially, the former mechanism does not alter the structure of different networks within a bacterium since the same suite of genes remains. In contrast, the latter does change network structure: the organism gains new genes that must be assimilated into networks controlling transcriptional regulation, metabolism, and transcription/translation.

**Figure 1:**
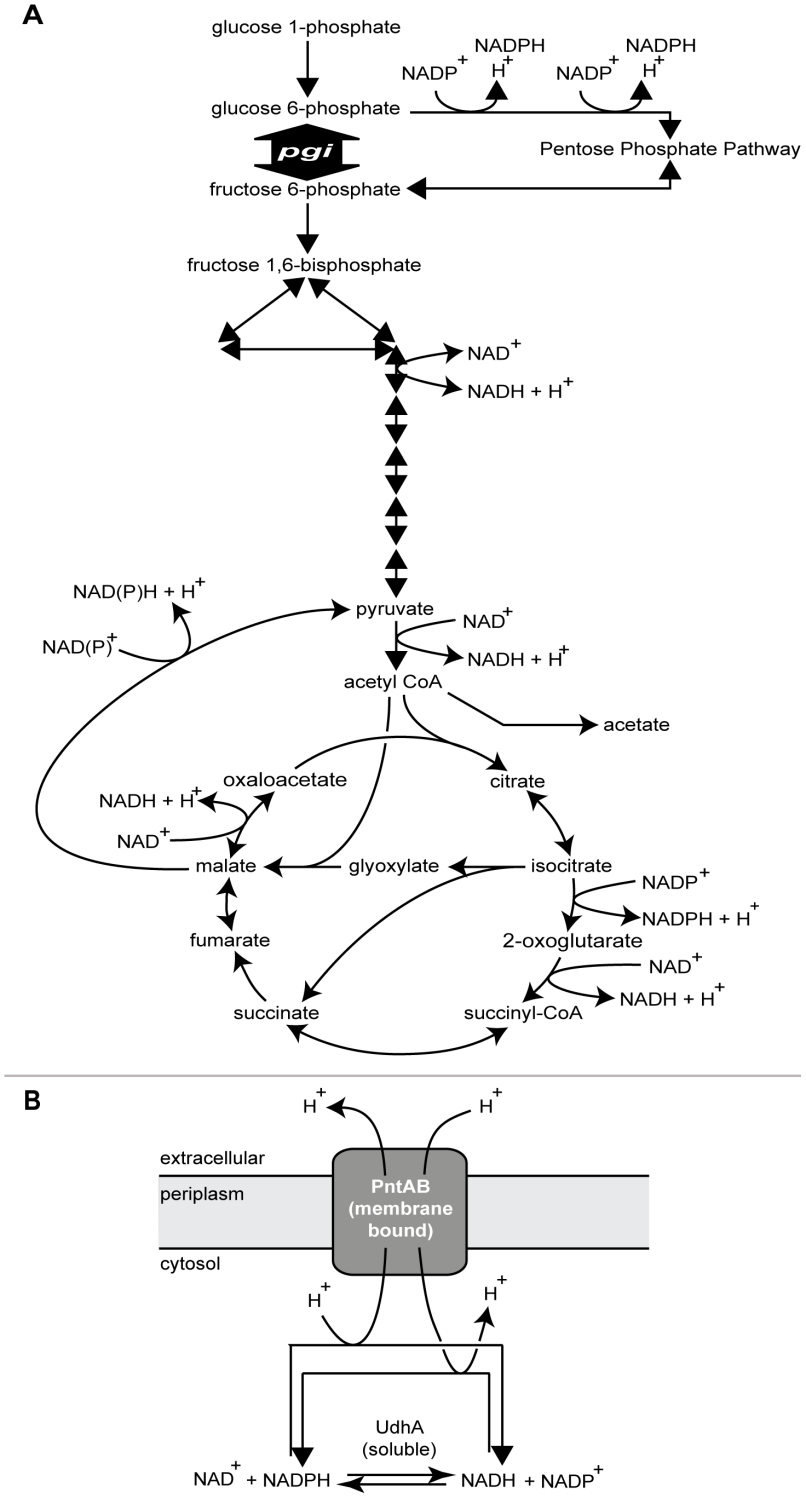
Role of *pgi*, *udhA*, and *pntAB* in cellular metabolism. A. The gene *pgi* catalyzes the isomerization of glucose 6-phosphate to fructose 6-phosphate in upper glycolysis. Removal of this gene forces glycolytic flux through the pentose phosphate pathway, creating a redox imbalance due to excess NADPH production. B. The genes *udhA* and *pntAB* catalyze the interconversion of NAD/NADH and NADP/NADPH. UdhA is a soluble protein whereas PntAB is membrane-bound.

Here we address an important related question: how does a bacterium adjust to the complete loss of a major gene product, not just to mutations in the gene? The answer to this question would provide insight into the plasticity of both bacterial genomes and the networks that emerge from the proteins they encode. We selected the gene *pgi* for study as it plays a major role in central metabolism by transcribing the enzyme that catalyzes the second step in glycolysis (Figure 1A). In *E. coli*, loss of *pgi* significantly alters the structure of its metabolic network by disabling the use of upper glycolysis, a situation that cripples growth (growth rate <20% of wild-type levels in glucose minimal media [13]) but does not kill the organism. *E. coli* Δ*pgi* mutants remain viable because glycolytic flux is rerouted through the pentose phosphate pathway (PPP) [14]–[15]; however, this introduces a redox imbalance problem since excess NADPH is produced which, in turn, perturbs a significant portion of the metabolic network [16]. Thus, *pgi* represents a good candidate gene to study mechanisms of compensation to gene loss.

To study how *E. coli* might overcome limitations imposed by the loss of *pgi* and consequent flux imbalances, we serially passed ten *E. coli* Δ*pgi* replicates for 50 days in glucose minimal media and characterized all evolved, endpoint clones at the phenotypic level through measurements of growth rates and rates of substrate uptake and secretion. We also assessed genotypic changes in nine of the strains through whole-genome resequencing using Nimblegen tiling arrays (all nine strains) and Illumina technology (three strains) to identify possible adaptive mutations that arose during evolution.

## Results

### The growth rates and glucose uptake rates of 50-day evolved Δ*pgi* mutants increased 3.6- and 2.6-fold on average, respectively, after adaptive evolution

**Figure 2:**
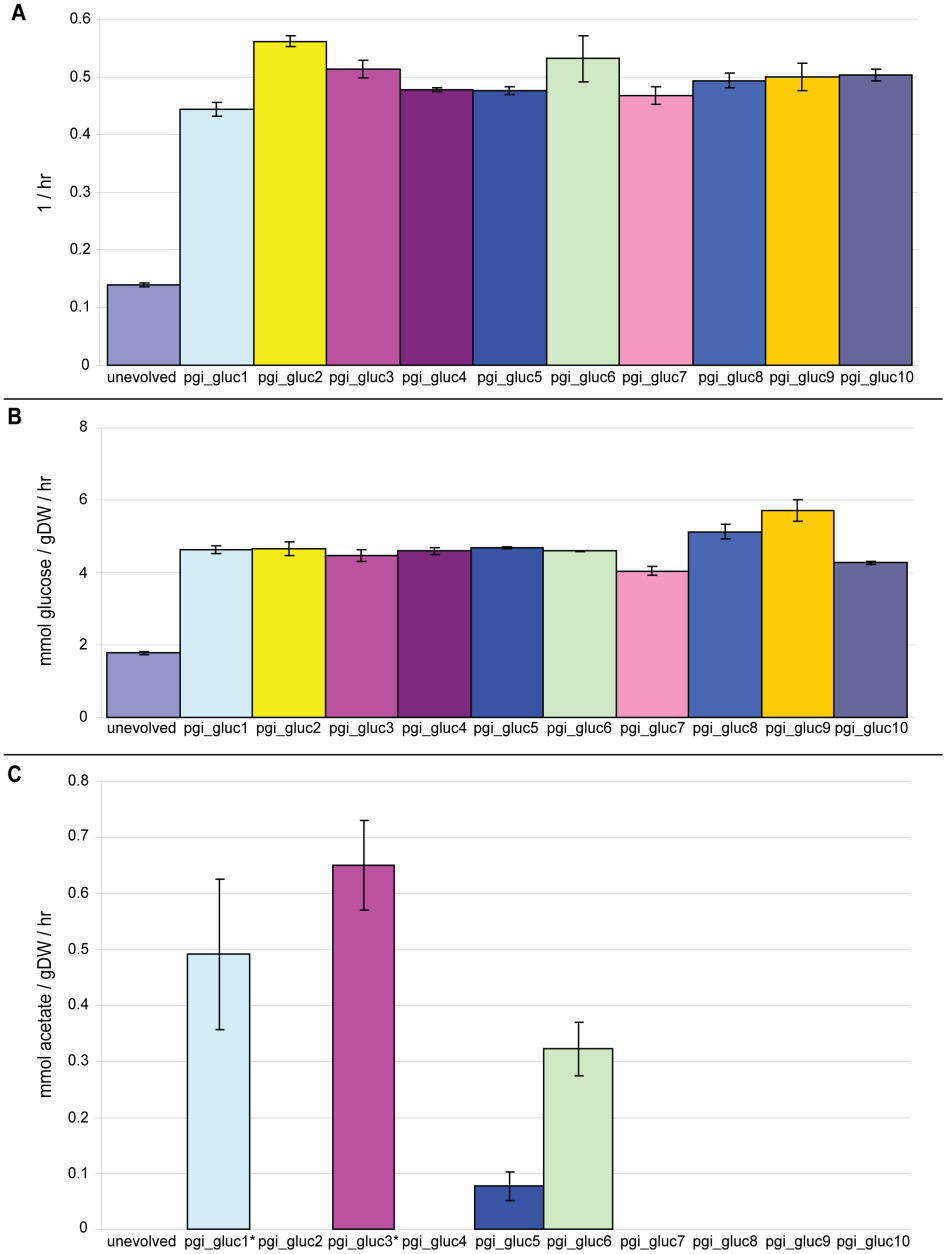
Growth rates, glucose uptake rates, and acetate secretion rates for unevolved and all evolved strains. A. Growth rates for the starting unevolved Δ*pgi* strain and all ten evolved strains after adaptive evolution. The growth rate of unevolved and 50-day evolved wild-type *E. coli* K12 MG1655 in the same medium was 0.69(0.0069 and 0.79(0.0092 hr-1. B. Glucose uptake rates for the unevolved (pgi strain and the ten evolved strains after adaptive evolution. Unevolved and 50-day evolved wild-type E. coli had a glucose uptake rate of 8.43(0.72 and 11.6(0.41 mmol/gDW/hour, respectively. C. Acetate secretion rates for the unevolved (pgi strain and the ten evolved strains after adaptive evolution. Unevolved and 50-day evolved wild-type E. coli had an acetate secretion rate of 12.8(8.87 and 5.61(0.24 mmol/gDW/hour, respectively. The symbol * indicates strains that also secrete formate (pgi_gluc1: 0.49(0.13 mmol/gDW/hour; pgi_gluc3: 0.22(0.02 mmol/gDW/hour). All error bars represent the standard deviation from three biological replicates. Abbreviations – gDW: gram dry weight; hr: hour.

All ten replicates converged to similar endpoint phenotypes after fifty days of serial passage and adaptive evolution in glucose M9 minimal media when assessed for changes in growth rates (Figure 2A) and glucose uptake rates (Figure 2B). These ten strains, as well as all other strains used in this study, are summarized in Table 1. On average, the growth rates for the ten strains exhibited a 3.6-fold increase over the starting unevolved Δ*pgi* strain to a final value of 0.50±0.03 hr^−1^. That growth rates of parallel replicates converge during adaptive evolution has been observed previously and thus seems to be a reproducible phenotypic outcome of such studies [17]. The glucose uptake rates for the evolved strains exhibited more variability; it increased 2.6-fold to a final average value of 4.68±0.46 mmol/gram dry weight/hour.

**Table 1:** Strains used in this study.

Strain	Characteristics	Source
Δ*pgi*	Starting strain for adaptive evolutions	Fong et al. [45]
pgi_gluc1	50-day evolved Δ*pgi* strain. Secretes acetate and formate	this study
pgi_gluc2	50-day evolved Δ*pgi* strain.	this study
pgi_gluc3	50-day evolved Δ*pgi* strain. Secretes acetate and formate	this study
pgi_gluc4	50-day evolved Δ*pgi* strain.	this study
pgi_gluc5	50-day evolved Δ*pgi* strain. Secretes acetate	this study
pgi_gluc6	50-day evolved Δ*pgi* strain. Secretes acetate	this study
pgi_gluc7	50-day evolved Δ*pgi* strain.	this study
pgi_gluc8	50-day evolved Δ*pgi* strain.	this study
pgi_gluc9	50-day evolved Δ*pgi* strain.	this study
pgi_gluc10	50-day evolved Δ*pgi* strain.	this study
KI2_rpoS	Δ*pgi* with in-frame duplication in *rpoS* at 595–603	this study
KI4_rpoS	Δ*pgi* with 1 base pair deletion in *rpoS* at position 841	this study
KI5_rpoS	Δ*pgi* with 1 base pair deletion in *rpoS* at position 850	this study
KI6_rpoS	Δ*pgi* with out-of-frame duplication in *rpoS* at 597–603	this study
KI7_rpoS	Δ*pgi* with c829t mutation in *rpoS*	this study
KI2_udhA	Δ*pgi* with *udhA* g(-64)a mutation	this study
KI2_pntA	Δ*pgi* with *pntA* c197a mutation	this study
KI2_RU	Δ*pgi* double knock-in strain with *rpoS* 595–603 duplication and *udhA* g(-64)a SNP	this study
KI2_RP	Δ*pgi* double knock-in strain with *rpoS* 595–603 duplication and *pntA* c197a SNP	this study
KI2_UP	Δ*pgi* double knock-in strain with *udhA* g(-64)a and *pntA* c197a mutations	this study
KI2_3KI	Δ*pgi* triple knock-in strain with *rpoS* 595–603 duplication and *udhA* g(-64)a and *pntA* c197a SNPs	this study

Knock-in strains that were constructed by introducing mutations identified during adaptive evolution back into the starting unevolved Δ*pgi* strain are designated “KI.” This designation is followed next by a number that corresponds to the evolved replicate on which the knock-in strain is based. The identity of the gene(s) containing the mutation follows last. Abbreviations – RU: *rpoS + udhA*; RP: *rpoS + pntA*; UP: *udhA + pntA*; 3KI: triple knock-in.

Three replicates of wild-type *E. coli* were also serially passed in glucose M9 for fifty days and assessed for changes in growth rate. The average initial growth rate was 0.69±0.0069 hr^−1^. The average growth rate after serial passage was 0.79±0.0092 hr^−1^, which constitutes a 1.1-fold increase. A separate study in which wild-type *E. coli* was evolved on glucose minimal media over 44 days reported slightly lower initial and final growth rates but a similar value for the fold increase over the evolutionary period [13].

### Adaptive evolution produced multiple, different endpoint clones as defined by their rates of metabolic by-product secretion

All ten strains evolved toward similar endpoint growth rates and glucose uptake rates but, interestingly, different metabolic functional states. Specifically, the ten strains could be stratified into three distinct groups based on their rates of acetate and formate secretion: those that secrete both acetate and formate, those that secrete acetate only, and those that secrete neither acetate nor formate (Figure 2C). Those that do secrete acetate do so at a rate about one-tenth lower than the acetate secretion rate for both unevolved wild-type *E. coli* (12.8±8.87 mmol acetate/gram dry weight/hour) and *E. coli* strains adapted to grow in glucose M9 for 50 days (5.61±0.24 mmol acetate/gram dry weight/hour) (Figure 2C). The parental Δ*pgi* clone did not secrete acetate.

### Two to five mutations were detected during the course of adaptation in the nine evolved Δ*pgi* strains that were sequenced

The mutations detected after the 50-day adaptive evolution period for the nine sequenced strains are summarized in Table 2. Mutations in *rpoS* were most common, appearing in six of nine endpoints. The *rpoS* mutation in pgi_gluc7 encodes a stop codon at that position; the *rpoS* mutations in pgi_gluc4, pgi_gluc5 and pgi_gluc6 likely result in truncated forms of the protein; the *rpoS* mutation in pgi_gluc3 is a SNP that results in a G279V change in the protein; and the *rpoS* mutation in pgi_gluc2 is an in-frame nine base pair duplication.

**Table 2:** Mutations detected in clones isolated from nine of the ten evolved Δ*pgi* strains isolated after 50 days of adaptive evolution.

	pgi_gluc1	pgi_gluc2	pgi_gluc3	pgi_gluc4	pgi_gluc5	pgi_gluc6	pgi_gluc7	pgi_gluc8	pgi_gluc10
*rpoA*			c854t			Δ944..963			t805c
*rpoB*	a3724c								
*rpoC*	c3520a								
*rpoS*		Dupl. (595–603)	g836t	Δ1bp (841)	Δ1bp (850)	Dupl. (597–603)	c829t		
*udhA*	a949g	g(−64)a	g(−64)t				g(−64)a	g(−64)a; g58a	
*pntA*		c197a					Δ680..690		
*pntB*				Δ1bp (430)					Dupl. (698–703)
*rep*	t242g								
*fabZ*	c167t								
*cpxR*			g614a	Δ4bp, 585..588					
*yfeH*				Δ1bp (297)					
*fruK*					c886t				
*rodA*							c263a		
*cyaA*								a1175c	
*bipA*								1bp Dupl. (960)	
*ispU*									t659g
Large indel		e14 prophage deletion							

All nine were sequenced using Nimblegen tiling arrays. Strains pgi_gluc1, pgi_gluc7, and pgi_gluc10 were also sequenced using first-generation Solexa (Illumina) technology. Strain pgi_gluc9 was not sequenced. Sanger sequencing was used to validate all reported mutations. Additionally, for all nine strains we sequenced the *rpoS*, *udhA*, *pntA* and *pntB* genes in their entirety using Sanger sequencing, not just specific regions indicated by Nimblegen or Solexa technologies to contain a mutation. The full length of these four genes was similarly sequenced in the three evolved wild-type (*rpoS* ^+^) replicates; no mutations were found at any position. No other genomic positions were sequenced in the evolved wild-type replicates. Abbreviations – bp: base pair; Dupl: duplication.

Mutations in the soluble transhydrogenase *udhA* [18] (six mutations in five strains) and membrane-bound transhydrogenase subunits *pntA* and *pntB* (two mutations each) were also common (Table 2). Loss of *pgi* directly perturbs both since they catalyze the oxidation and reduction of NAD(P)/NAD(P)H (Figure 1B). Interestingly, the same position, –64 base pairs (bp) upstream from the annotated *udhA* transcription start site, was mutated in four of the five strains with *udhA* mutations. This mutation was the only one that developed outside of a coding region. On the other hand, three of the four *pntAB* mutations likely result in truncated, nonfunctional proteins: the mutation in pgi_gluc2 *pntA* is a nonsense mutation while the pgi_gluc7 *pntA* and pgi_gluc4 *pntB* mutations truncate the proteins from 510 to 265 and 462 to 154 amino acids, respectively. The pgi_gluc10 *pntB* mutation extends the protein length by two amino acids: two additional alanines are added to a region where four alanines are already present, resulting in six consecutive alanine residues.

Whereas the evolved Δ*pgi* replicates frequently developed mutations in *rpoS*, *udhA*, and *pntAB*, no mutations could be detected in these four genes in the three wild-type (*pgi* ^+^) *E. coli* replicates evolved under the same growth conditions. This finding suggests that the mutations in *rpoS*, *udhA* and *pntAB* stemmed from adaptation to loss of *pgi* rather than other possible selection pressures such as the growth medium, a phenomenon that occurs when wild-type *E. coli* is cultured in minimal media containing glycerol or lactate as the sole carbon source [1], [4].

Another noteworthy observation is the frequency with which genes involved in global regulation were mutated – at least one in all nine strains. Besides *rpoS*, other genes that modulate transcription of multiple loci and that developed mutations included *rpoA*, *rpoB*, *rpoC*, *cyaA* and *cpxR* (Table 2). This result implies that adaptation required global, network-level changes to transcriptional regulation and metabolism, which is emerging as a general, recurring theme across multiple organisms. For example, *E. coli* strains adapted to grow on glycerol as the sole carbon source often develop causative mutations in RNAP [1]; a mutation in the transcription factor *Spt15p* confers greater ethanol tolerance in yeast [19]; and a mutation in the sensor kinase of a two-component signal transduction system enhanced transition to invasive infection and virulence in a mouse model of Group A Streptococcus pathogenesis [8].

**Figure 3:**
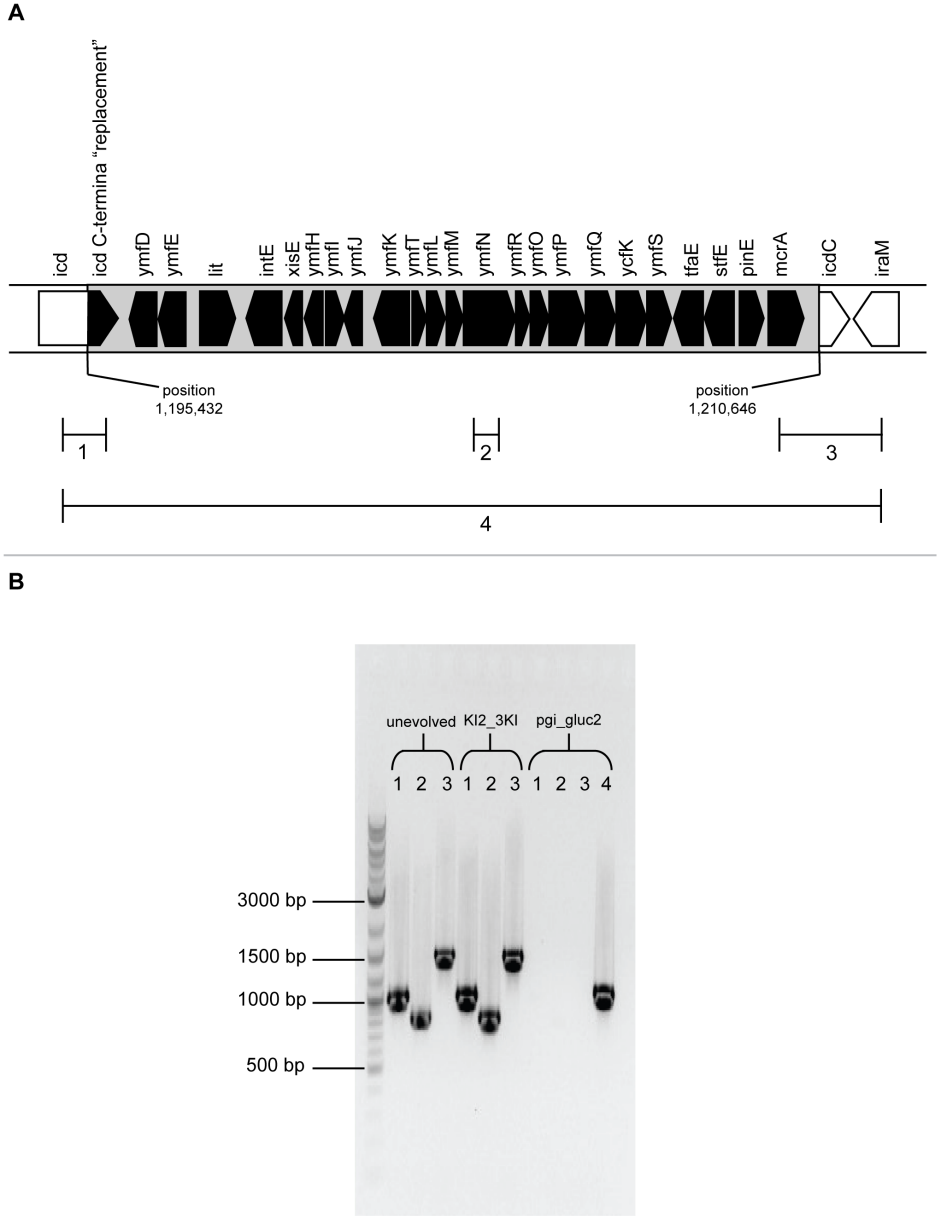
Loss of e14 prophage in pgi_gluc2. A. Structure and location of the e14 prophage. It is integrated within the icd gene in the E. coli chromosome at position 1,195,432 to position 1,210,646. B. PCR analysis of the unevolved (pgi, pgi_gluc2 triple knock-in (KI2_3KI) and evolved pgi_gluc2 strains confirms loss of the e14 prophage in pgi_gluc2. Numbers 1 through 4 correspond to PCR amplification regions as indicated in the top panel. Regions 1 and 3 both span terminal segments of the integrated prophage and adjacent chromosomal DNA. Region 2 spans a segment wholly within the prophage. Region 4 spans the integration site and is amplified using the left primer from Region 1 and the right primer from Region 3.

Lastly, we detected one large indel: loss of the 15.4 kbp e14 prophage in pgi_gluc2. Its deletion was initially suggested through both analysis of the resequencing data and an optical map for this strain. It was subsequently confirmed by PCR analysis of the ends of the prophage and flanking regions (Figure 3). We could not detect large indels in the resequencing data for any other strain; however, we cannot rule out the possibility that they might still be present, especially transposition of mobile elements such as insertion sequences (IS elements), because of limitations inherent in the two short-read resequencing technologies employed here. IS elements in particular have been shown to translocate frequently in *E. coli* when it undergoes adaptive evolution under a wide variety of conditions [20]–[23]. If the raw sequences do not assemble into contigs large enough to sufficiently span the mobile element and flanking regions, they can be difficult to map accurately to possible new locations in the genome.

### Most mutations in *rpoS* likely result in nonfunctional proteins and none confer increased growth rate when present alone

**Figure 4:**
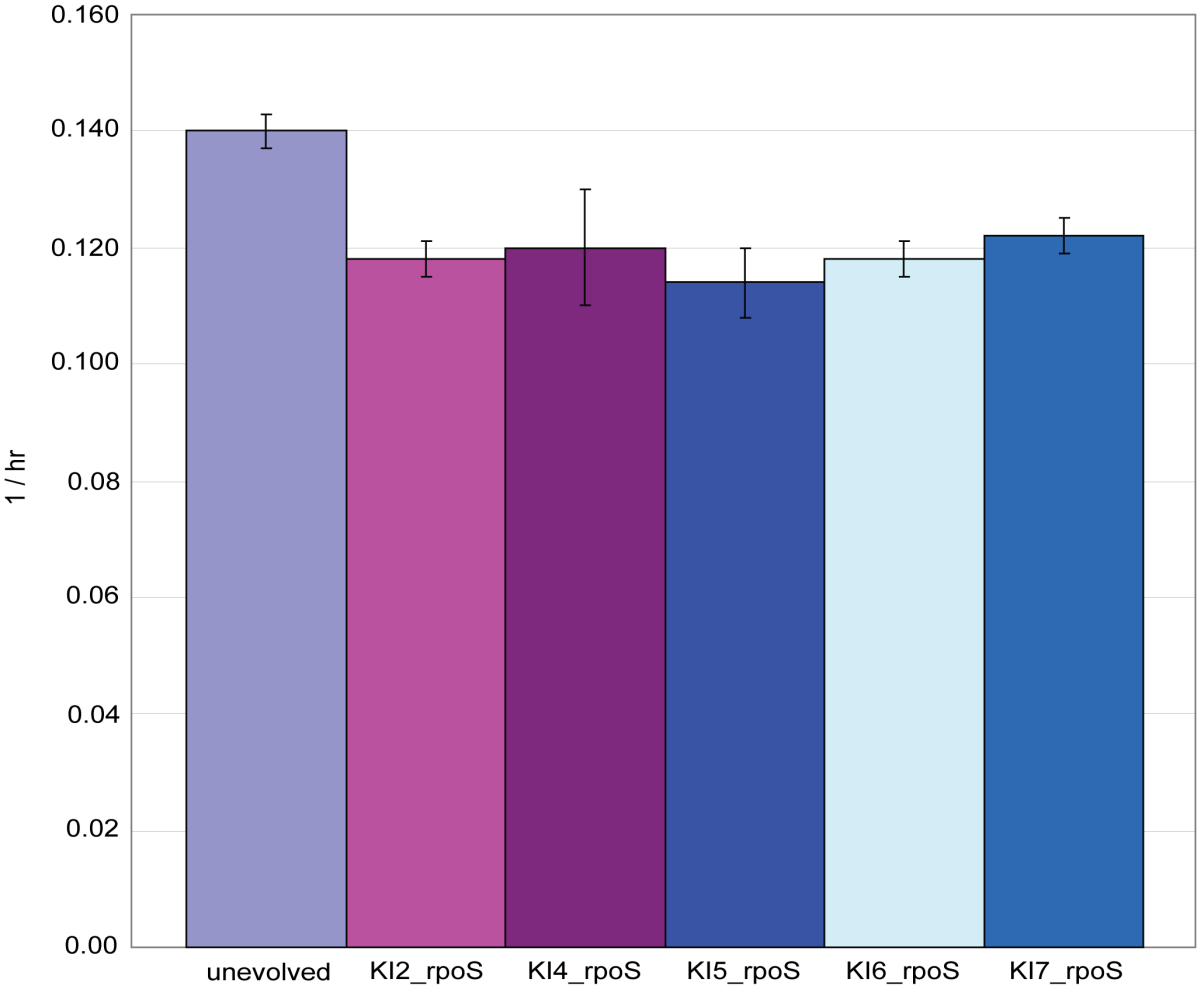
Growth rates for five *rpoS* knock-in strains. The strains were constructed by introducing five of the six *rpoS* mutations detected after adaptive evolution back into the starting unevolved Δ*pgi* strain through site-directed mutagenesis. A similar knock-in strain containing the pgi_gluc3 *rpoS* mutation was not constructed. Growth rate data for the starting unevolved Δ*pgi* strain is also shown for comparison. Error bars represent the standard deviation from three biological replicates. A full description for the strain abbreviations can be found in Table 1. Abbreviations – hr: hour.

The high frequency of *rpoS* mutations prompted us to investigate whether this set of mutations had any impact on the growth rate increases seen in the evolved Δ*pgi* strains. When five of the six *rpoS* mutations were introduced into the chromosome of the starting unevolved Δ*pgi* clone, none of the five knock-in strains displayed an increase in growth rate (Figure 4). Surprisingly, all had growth rates slightly lower than that of the unevolved clone, indicating that they are neutral to slightly deleterious (Student's t-test, P<0.01 for all five). We then investigated indirectly whether the *rpoS* mutants still encoded functional proteins by flooding single colonies of each of the five knock-in strains with hydrogen peroxide. Vigorous bubbling occurs if *rpoS* is functional due to RpoS control of *katE* expression [24]. For comparison, we also performed this assay on the ten evolved strains, the starting unevolved Δ*pgi* strain and a Δ*rpoS* mutant obtained from the Keio collection [25]. The five knock-in strains, the six evolved strains harboring *rpoS* mutations and, interestingly, one of the evolved strains that did not have an *rpoS* mutation (pgi_gluc1) all exhibited reduced bubbling upon contact with hydrogen peroxide (Table S1). The pgi_gluc1 result indicates that other genes besides *rpoS* can control *katE* expression. As expected, the Δ*rpoS* mutant also exhibited reduced bubbling. In contrast, bubbling remained vigorous for two of the three evolved strains (pgi_gluc8 and pgi_gluc10) that did not harbor an *rpoS* mutation. It therefore appears that the majority of *rpoS* mutations result in non-functional proteins and that they do not have a significant impact on growth rates when present alone.

### The three mutations in pgi_gluc2 display both positive and negative epistatic interactions

**Figure 5:**
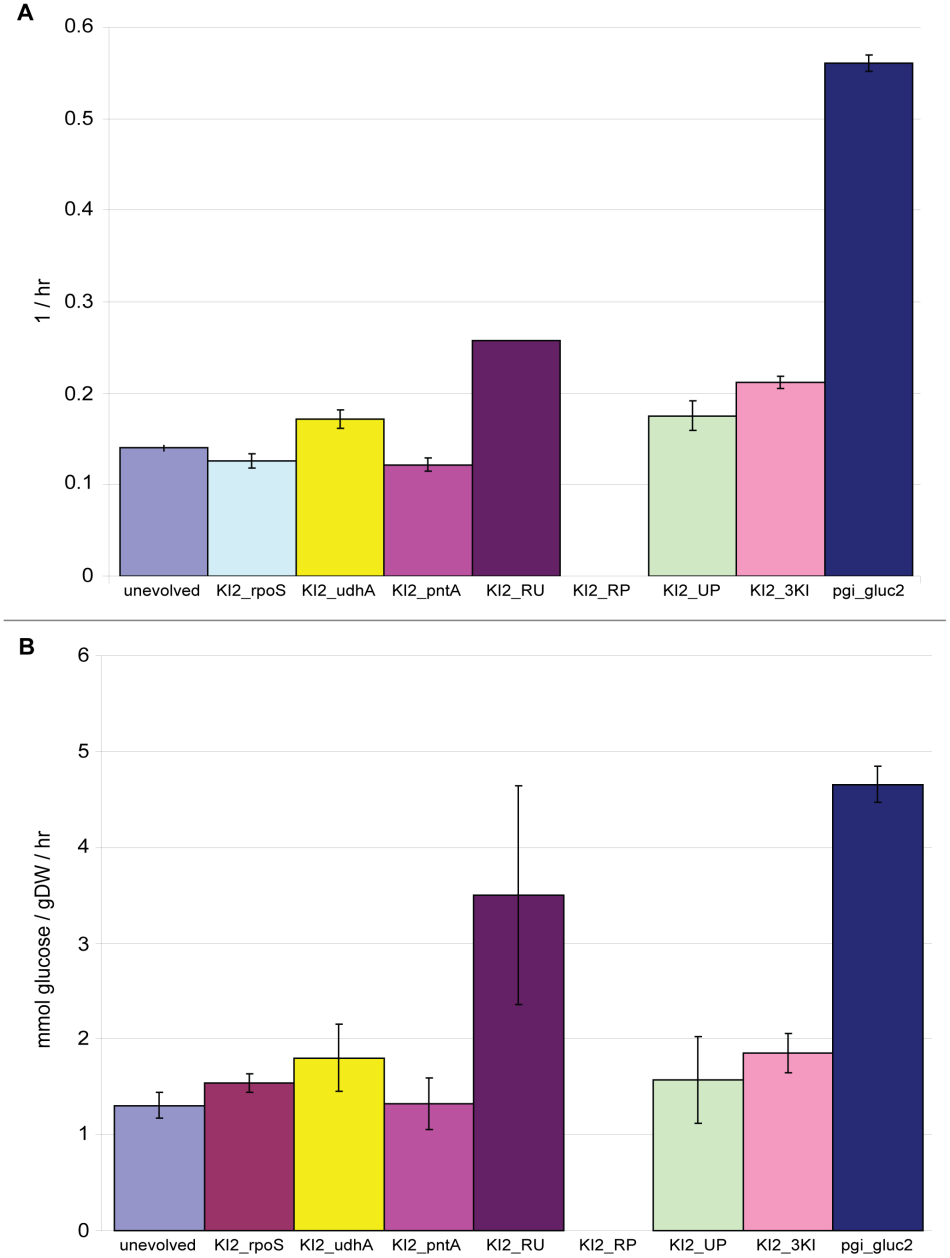
Growth rates and glucose uptake rates for pgi_gluc2 single, double, and triple knock-in strains. A. Growth rates for the three single, three double and triple knock-in strains constructed based on the three mutations that appeared in pgi_gluc2. The growth rates for the starting unevolved Δ*pgi* strain and the evolved pgi_gluc2 strain are also shown for comparison. B. Corresponding glucose uptake rates for the seven knock-in strains. Data for the unevolved and evolved strains are again shown for comparison. Error bars for both represent the standard deviation from three biological replicates. A full description for the strain abbreviations can be found in Table 1. Abbreviations – gDW: gram dry weight; hr: hour.

We next investigated how the *rpoS*, *udhA* and *pntA* mutations in pgi_gluc2 influence its growth rate by constructing all three single knock-ins, all three double knock-ins, and the triple knock-in from the starting unevolved Δ*pgi* strain. Like *rpoS*, the *pntA* single knock-in strain grew slightly more slowly than the unevolved Δ*pgi* strain (Figure 5). This slight growth rate reduction is perhaps expected since the c197a mutation in *pntA* changes the codon triplet at that position from a serine to a stop codon, truncating the protein from 510 to 65 amino acids and likely rendering it non-functional. The *udhA* mutation, on the other hand, does impact the growth rate; the *udhA* single knock-in strain grew at a rate 1.4 times faster than the *rpoS* and *pntA* single knock-in strains (*P*<0.001).

Construction of the three double knock-in strains revealed both positive and negative epistasis among the three mutations. There is positive epistasis between the *rpoS* and *udhA* mutations. Since the *rpoS* single knock-in strain has a growth rate very similar to that of the unevolved Δ*pgi* clone, one would expect the growth rate of the *rpoS + udhA* double knock-in strain to closely mimic that of the single *udhA* knock-in strain if the two mutations were independent. Instead, the double knock-in had a growth rate 1.8 and 1.5 times greater than that of the unevolved and the single *udhA* knock-in strains, respectively (Figure 5). We conclude from these data that, in addition to *udhA*, the *rpoS* mutation is causal. In contrast, there is negative epistasis between the *rpoS* and *pntA* mutations. This double knock-in strain grew well overnight as a pre-culture in LB medium, but failed to grow after washing twice and transferring to glucose M9 media, even after five days incubation. Lastly, the *udhA* and *pntA* mutations do not show any apparent epistasis since the growth rate for this double knock-in strain was essentially identical to that of the *udhA* single knock-in strain.

The growth rate for the triple knock-in strain was only 0.21 hr^−1^, less than half that of the evolved pgi_gluc2 strain (Figure 5). This finding implies that deletion of the e14 prophage likely contributes significantly to adaptation to loss of *pgi* in this genetic background.

## Discussion

We have investigated how *E. coli* overcomes the loss of *pgi*, a challenge that forces glycolytic flux through the pentose phosphate pathway and creates a redox imbalance in the cell. The data presented here indicate that adapted Δ*pgi* mutants accomplish this task through mutations in key genes, in particular *rpoS* and the transhydrogenases *udhA* and *pntAB*, that suppress the bacterial stress response and likely ameliorate the redox imbalance, respectively. Multiple alternative phenotypes arise from these mutations as defined by their rates of acetate and formate secretion.

One explanation for the high frequency of *rpoS* mutations is subordination of the *rpoS-*controlled stress response in favor of *rpoD-*controlled maximization of nutrient uptake and utilization. The *rpoS* gene encodes a sigma factor most active during stationary phase but which also affects the expression of about fifty genes during log phase [26]. It controls general stress response in *E. coli* and related bacteria [27]–[28] but does so at the considerable expense of reduced expression of *rpoD*-controlled housekeeping genes [29]. Perhaps most importantly, *rpoD* also controls expression of genes involved in nutrient scavenging in nutrient-limited environments [29]. This trade-off, designated stress protection and nutritional competence (SPANC) [30]–[31], consequently creates a conflict between the hunger and stress responses in *E. coli* since both sigma factors cannot simultaneously bind RNAP. Since the growth media for the ten Δ*pgi* and three wild-type *E. coli* evolutions all contained the same initial glucose concentration (2 g/L), the greatly reduced growth rate of the unevolved Δ*pgi* clone compared to wild-type *E. coli* demonstrates clearly that the former does not convert nutrients into biomass optimally. This growth rate reduction, which imposes a stress, is likely sufficient to induce high levels of RpoS in the unevolved Δ*pgi* strain and shift the SPANC balance from metabolism to stress. The *rpoS* mutations that emerged in six of the evolved strains, all of which reduce functionality of the protein as indicated by the peroxidase assay (Table S1), would shift the balance from stress back to metabolism in these six to allow for greater *rpoD-*controlled nutrient acquisition and consequent faster growth.

There are several other notable mutations besides those in *rpoS*. As mentioned, the –64 position upstream of the *udhA* transcription start site was mutated in four out of five strains. Because u*dhA* plays a major role in oxidizing NADPH with NAD during conditions of excess NADPH and its overexpression increases the growth rate of unevolved Δ*pgi* mutants [32], we speculate that this mutation upregulates *udhA* expression. In contrast, three of the four *pntAB* mutations likely reduce or abolish PntAB function since they produce peptides that are severely truncated. Decreasing PntAB function prevents the redox imbalance condition caused by loss of *pgi* from worsening: ^13^C-flux measurements show that PntAB produces 35–45% of the NADPH in *E. coli* during standard batch growth on glucose [32]. The mutations in *udhA* and *pntAB* detected here thus support previous findings [32] that the two transhydrogenases have divergent functions despite their shared ability to catalyze the interconversion of NAD/NADH and NADP/NADPH reversibly. Whereas UdhA plays a major role in oxidizing NADPH with NAD, the membrane-bound PntAB plays a major role in reoxidizing NADH with NADP. The pgi_gluc1 *fabZ* mutation (missense; P167L) is noteworthy because *fabZ* is involved in fatty acid biosynthesis, a process that utilizes NADPH. This raises the possibility that NADPH overabundance in Δ*pgi* mutants drives excessive fatty acids biosynthesis, translating the redox imbalance into an imbalance in fatty acid production. The *fabZ* mutation in pgi_gluc1 might help to alleviate this imbalance by decreasing flux through this pathway.

The observation that the pgi_gluc2 triple knock-in strain could not reproduce the same growth rate as the evolved strain implies that loss of the e14 prophage plays a role in adaptation. This prophage lies at position 1,195,432 bp to 1,210,646 bp [33] on the chromosome and contains 24 putative ORFs, many of which have unknown function [34]. Exposure to UV radiation consistently activates excision of this element [35]–[36], probably through induction of the SOS response [37]. This observation implies that the SOS response was probably induced at some point during adaptation to loss of *pgi* in pgi_gluc2. Deletion of e14 might contribute to adaptation to loss of *pgi* in several ways. First, e14 contains several genes whose presence/absence likely affects metabolism, for example a toxin encoded by *kil* that kills the cell in the absence of its repressor, which is also encoded by e14 [34], [38]. Second, deletion of e14 introduces ten synonymous and two non-synonymous mutations into the amino acid sequence of isocitrate dehydrogenase (*icd*), a key metabolic enzyme [39]. Although none of these mutations alters the specific activity of the protein in cell-free experiments [39], they could exert an effect *in vivo* through their potential impact on translation efficiency.

Acetate secretion rates are zero to approximately ten times lower in the ten evolved Δ*pgi* replicates compared to unadapted and glucose-adapted wild-type *E. coli* (Figure 2C), which implicates a connection between acetate metabolism and loss of *pgi*. We speculate that this connection stems from a need to maximize flux through the TCA cycle in *E. coli* Δ*pgi* mutants. It is well known that wild-type *E. coli* secretes acetate during aerobic growth on glucose [40]–[41], a phenomenon known as overflow metabolism [42], when acetyl-CoA converts to acetate rather than enter the TCA cycle. On the other hand, the loss of *pgi* forces flux normally occurring in upper glycolysis through the pentose phosphate pathway, reducing flux through lower glycolysis [32] and presumably reducing the amount of acetyl-CoA that enters the TCA cycle relative to that in wild-type *E. coli*. Minimizing by-product secretion due to overflow metabolism would therefore serve to maximize the reduced amount of flux flowing through lower glycolysis that enters the TCA cycle. The regulation of acetate metabolism, however, is a complex process [43]–[44], and other factors could be involved.

Besides contrasting acetate secretion profiles, we hypothesize that evolved Δ*pgi* strains utilize the glyoxylate shunt whereas their evolved wild-type (*pgi* ^+^) counterparts do not since this shunt is already active in unevolved *E. coli* lacking *pgi* [32]. Routing flux partly through the glyoxylate shunt rather than the full TCA cycle would avoid exacerbating the redox imbalance problem because NAD(P)H biosynthesis normally arising from the conversion of isocitrate to α-ketoglutarate (NADPH) and α-ketoglutarate to succinyl-CoA (NADH) would not occur.

In conclusion, we have examined the robustness of the *E. coli* metabolic network and the genetic basis for adaptation to the loss of *pgi* through adaptive evolution, resequencing and phenotypic assays. Two to five mutations were detected after adaptation and were most frequently located in the alternative sigma factor *rpoS*, the soluble transhydrogenase *udhA*, and the membrane-bound transhydrogenase *pntAB*. The genetic and biochemical data collected here paints a picture in which one general mechanism through which *E. coli* adapts to loss of *pgi* and manages the redox imbalance problem occurs via 1) favoring *rpoD-*controlled hunger response over *rpoS-*controlled stress response, and 2) curtailing (and possibly eliminating) PntAB function to lessen PntAB-catalyzed NADPH biosynthesis. We also found evidence showing that adaptive evolution can lead to multiple, alternative phenotypes within the evolutionary landscape, defined in this study as a difference in byproduct secretion rates. Looking forward, adaptation to loss of *pgi*, and perhaps to loss of other metabolic genes, might constitute a model system to investigate the link between genotype and phenotype through creation of additional knock-in mutants and examining the effect of each mutation on metabolic flux. Such studies would further highlight the plasticity of the bacterial genome and cellular networks as well as delineate mechanisms through which bacteria adapt to genetic perturbations.

## Methods

### Strains

The strains used in this study are summarized in Table 1. The starting strain for all adaptively evolved replicates was an *E. coli* Δ*pgi* strain constructed from wild-type *E. coli* K12 MG1655 (ATCC, Manassas, VA) as described previously [45]. Whole-genome resequencing identified three base pair changes in the MG1655 Δ*pgi* strain utilized here that were not present in the sequenced MG1655 strain: a c→g mutation in *ybeB* (genomic position 667965), an a→g mutation in *ylbE_1* (genomic position 547694) and a c→a mutation in *nupC* (genomic position 2511373). Sanger sequencing was used to confirm these three mutations. Strains pgi_gluc1 through pgi_gluc10 were all generated during this study through adaptive evolution of the starting *E. coli* Δ*pgi* strain. Knock-in strains in which mutations detected after adaptive evolution were introduced into the starting unevolved Δ*pgi* strain were created using the method of Tischer et al. [46] with the following modifications: 1) pKD46, a temperature-sensitive plasmid that carries bacteriophage λ *red* genes (γ, β, and *exo*) under the control of the arabinose-inducible P*_araBAD_* promoter [47], was first electroporated into cells to be transformed and subsequently used for the first Red recombination step, 2) pKD13 [47] was used to amplify the kanamycin resistance gene instead of pACYC177, and 3) pACBSR, which contains a chloramphenicol-resistance selection marker and encodes I-SceI and the λ *red* system both under the control of an arabinose-dependent promoter [48], was used for the second Red recombination step. The primers used to construct the knock-in strains can be found in Table S2.

### Growth media

The starting Δ*pgi* clone was maintained and propagated in Luria-Bertrani (LB) broth (EMD Chemicals, Gibbstown, NJ). It was not exposed to glucose M9 minimal medium prior to this study. The composition of the glucose M9 was: dextrose (2 g/L), CaCl_2_ (100 µM), MgSO_4_ (200 mM), Na_2_HPO_4_ (13.6 g/L), KH_2_PO_4_ (6 g/L), NaCl (1 g/L), NH_4_Cl (2 g/L) and trace elements (500 µL). The trace element solution consisted of (per liter): FeCl_3_•6H_2_O (16.67 g), ZnSO_4_•7H_2_O (0.18 g), CuCl_2_•2H_2_O (0.12 g), MnSO_4_•H_2_O (0.12 g), CoCl_2_•6H_2_O (0.18 g) and Na_2_EDTA•2H_2_O (22.25 g).

### Adaptive evolution protocol

The adaptive evolution protocol used here has been described previously [45]. To summarize, all ten Δ*pgi* replicates and the three wild-type replicates were serially passed daily in 250 mL glucose M9 minimal medium in 500 mL Erlenmeyer flasks for 50 days at 37°C using magnetic stir bars for aeration. The volume transferred was adjusted each day to account for changes in growth rate in order to maintain cultures in prolonged exponential phase growth and thereby avoid entry into stationary phase. On Day 50, an aliquot of each replicate was streaked out on LB plates containing 20 g/L bacteriological agar (Sigma-Aldrich, St. Louis, MO) and incubated at 37°C for 24 hours. A well-isolated colony was then selected and suspended in LB broth containing 25% glycerol and stored long-term at −80°C.

### Whole-genome resequencing

We isolated genomic DNA using a Qiagen DNeasy kit (QIAGEN, Valencia, CA) from the same single-colony sample collected for long-term storage. Whole-genome sequencing was then carried out using two platforms, Nimblegen hybridization-based tiling arrays [49] and Illumina technology. Nimblegen also provided data analysis capabilities [49] for identification of possible mutations, while Solexa results were analyzed using a software program developed in-house that counts the total number of each base for a given position and compares the consensus call to the reference *E. coli* genome. All reported mutations were confirmed by PCR amplification of the surrounding DNA region and Sanger sequencing. Additionally, we also used Sanger sequencing to sequence the full-length *rpoS*, *udhA*, *pntA*, and *pntB* genes for the nine resequenced, evolved Δ*pgi* strains and the three evolved wild-type *E. coli* replicates, not just regions immediately surrounding mutations reported by Nimblegen and/or Illumina. For the evolved wild-type replicates, no other genomic positions were sequenced besides the full-length genes for these four. The list of primers used for both PCR amplification and Sanger sequencing is given in Table S3. The optical map for pgi_gluc2 was provided as a service by OpGen, Inc. (Gaithersburg, MD) using NcoI as the restriction enzyme.

### Growth rate and substrate uptake/secretion rate measurements

Overnight pre-cultures of each strain grown in LB medium were spun down and washed twice with glucose M9 minimal medium, after which they were used to inoculate 500 mL Erlenmeyer flasks containing 250 mL glucose M9 in triplicate. These flasks were incubated overnight in an air incubator maintained at 37°C using magnetic stir bars for aeration, conditions which were identical to those used during the adaptive evolutions. The next day an inoculum of each replicate was transferred to a new 500 mL Erlenmeyer flask again containing 250 mL of glucose M9 such that the optical density at 600 nm (OD600) had been reduced to 0.005. These flasks were then placed in a water bath maintained at 37°C. Magnetic stir bars were again used for aeration.

Once the OD600 reached 0.05 and then continuing periodically thereafter, the optical density of each sample was recorded and culture media were collected and filtered through 0.22 µm membranes. HPLC analysis (Waters, Milford, MA) was then carried out on the filtered media using a Bio-Rad Aminex HPX-87H ion exclusion column (300 mm×7.8 mm) with 5 mM H_2_SO_4_ as the mobile phase at a flow rate of 0.5 mL/min and temperature of 45°C.

## Supporting Information

Table S1 Indirect assessment of RpoS activity utilizing the peroxidase assay [19]. Colonies with functional RpoS exhibit vigorous bubbling when they come into contact with hydrogen peroxide via a mechanism based on *rpoS* control of *katE* expression. We defined vigorous bubbling (v) as bubble formation occurring within five seconds after contact with hydrogen peroxide; medium bubbling (m) as bubble formation occurring between five and ten seconds after contact; and slight bubbling (s) as bubble formation occurring ten seconds after contact. This assay was performed 24 and 48 hours after colonies had been inoculated onto LB plates. Abbreviations: repl - replicate.
(0.06 MB DOC)

Table S2 Primers used to introduce mutations detected after adaptive evolution back into the starting unevolved Δ*pgi* strain according to the method of Tischer et al [46].
(0.04 MB DOC)

Table S3 Primers used for Sanger sequencing to confirm mutations reported by Nimblegen and Illumina sequencing technologies. The *rpoS*, *udhA*, *pntA*, and *pntB* genes were sequenced in their entirety in all nine evolved strains using Sanger sequencing, not just regions immediately surrounding reported mutations. Primers were designed for this purpose by dividing the four genes into regions of approximately 800–900 base pairs with an overlap between regions of approximately 100 base pairs.
(0.06 MB DOC)
