# Supplementary material for: Genetic Basis of Growth Adaptation of Escherichia coli after Deletion of pgi, a Major Metabolic Gene
Source: PLoS Genet. 2010 Nov 4;6(11):e1001186. doi: 10.1371/journal.pgen.1001186 (PMC2973815; doi:10.1371/journal.pgen.1001186)
Supplement: Table S1 — Indirect assessment of RpoS activity utilizing the peroxidase assay [19]. Colonies with functional RpoS exhibit vigorous bubbling when they come into contact with hydrogen peroxide via a mechanism based on rpoS control of katE expression. We defined vigorous bubbling (v) as bubble formation occurring within five seconds after contact with hydrogen peroxide; medium bubbling (m) as bubble formation occurring between five and ten seconds after contact; and slight bubbling (s) as bubble formation occurring ten seconds after contact. This assay was performed 24 and 48 hours after colonies had been inoculated onto LB plates. Abbreviations: repl - replicate. (0.06 MB DOC) [file pgen.1001186.s001.doc]

**Table S1. Indirect assessment of RpoS activity utilizing the peroxidase assay [19].**

|  | 24 hours | | | 48 hours | | |
| --- | --- | --- | --- | --- | --- | --- |
| Strain | repl. 1 | repl. 2 | repl. 3 | repl. 1 | repl. 2 | repl. 3 |
| unevolved Δ*pgi* | v | v | v | v | v | v |
| Δ*rpoS* | s | s | s | s | s | s |
| pgi_gluc1 | m | m | m | m | m | m |
| pgi_gluc2 | m | m | m | m/v | m/v | m/v |
| pgi_gluc3 | s | s | s | s | s | s |
| pgi_gluc4 | s | s | s | s/m | s/m | s/m |
| pgi_gluc5 | s | s | s | s | s | s |
| pgi_gluc6 | s | s | s | s | s | s |
| pgi_gluc7 | s | s | s | s | s | s |
| pgi_gluc8 | v | v | v | v | v | v |
| pgi_gluc9 | v | v | v | v | v | v |
| pgi_gluc10 | v | v | v | v | v | v |
| 3KI | s | m | m | m | m | m |
| KI2_rpoS | m | m | m | m | m | m |
| KI4_rpoS | s/m | s/m | s/m | s | s | s |
| KI5_rpoS | s | s | s | s | s | s |
| KI6_rpoS | s | s | s | s | s | s |
| KI7_rpoS | s | s | s | s | s | s |

Colonies with functional RpoS exhibit vigorous bubbling when they come into contact with hydrogen peroxide via a mechanism based on *rpoS* control of *katE* expression. We defined vigorous bubbling (v) as bubble formation occurring within five seconds after contact with hydrogen peroxide; medium bubbling (m) as bubble formation occurring between five and ten seconds after contact; and slight bubbling (s) as bubble formation occurring ten seconds after contact. This assay was performed 24 and 48 hours after colonies had been inoculated onto LB plates. Abbreviations: repl – replicate.
