# Supplementary material for: Genetic Basis of Growth Adaptation of Escherichia coli after Deletion of pgi, a Major Metabolic Gene
Source: PLoS Genet. 2010 Nov 4;6(11):e1001186. doi: 10.1371/journal.pgen.1001186 (PMC2973815; doi:10.1371/journal.pgen.1001186)
Supplement: Table S2 — Primers used to introduce mutations detected after adaptive evolution back into the starting unevolved Δpgi strain according to the method of Tischer et al [46]. (0.04 MB DOC) [file pgen.1001186.s002.doc]

**Table S2. Primers used to introduce mutations detected after adaptive evolution back into the starting unevolved Δ*pgi* strain according to the method of Tischer et al. [46].**

| Strain | Gene | Left primer | Right primer |
| --- | --- | --- | --- |
| pgi_gluc2 | *rpoS* | ACCATGAACCAAGTGCGGAAGAGATCGCAGAGCAAGCAGAGCAACTGGATAAGCCAGTTGATTAGGGATAACAGGGTAATATTCCGGGGATCCGTCGACC | TTAAGACGAAGCATACGGCTGACGTCATCAACTGGCTTATCCAGTTGCTCTGCTTGCTCTGCGATCTCTTCCGCACTTGGGTGTAGGCTGGAGCTGCTTC |
| pgi_gluc3 | *rpoS* | AGCTGAACGCCAAACAGCGTGAAGTGCTGGCACGTCGATTCGTTTTGCTGGGGTACGAAGCGTAGGGATAACAGGGTAATATTCCGGGGATCCGTCGACC | TTTGCTGGGGTACGAAGCGGCAACACTGGAAGATGTAGGTCGTGAAATTGGCCTCACCCGACGAATCGACGTGCCAGCACGTGTAGGCTGGAGCTGCTTC |
| pgi_gluc4 | *rpoS* | AACGCCAAACAGCGTGAAGTGCTGGCACGTCGATTCGGTTTGTGGGGTACGAAGCGGCAACATAGGGATAACAGGGTAATATTCCGGGGATCCGTCGACC | GTTCACGGGTGAGGCCAATTTCACGACCTACATCTTCCAGTGTTGCCGCTTCGTACCCCACAAACCGAATCGACGTGCCAGTGTAGGCTGGAGCTGCTTC |
| pgi_gluc5 | *rpoS* | CAGCGTGAAGTGCTGGCACGTCGATTCGGTTTGCTGGGGTACAAGCGGCAACACTGGAAGATTAGGGATAACAGGGTAATATTCCGGGGATCCGTCGACC | GGCGAACACGTTCACGGGTGAGGCCAATTTCACGACCTACATCTTCCAGTGTTGCCGCTTGTACCCCAGCAAACCGAATCGTGTAGGCTGGAGCTGCTTC |
| pgi_gluc6 | *rpoS* | GGACCATGAACCAAGTGCGGAAGAGATCGCAGAGCAAAGAGCAACTGGATAAGCCAGTTGATTAGGGATAACAGGGTAATATTCCGGGGATCCGTCGACC | CGTTAAGACGAAGCATACGGCTGACGTCATCAACTGGCTTATCCAGTTGCTCTTTGCTCTGATCTCTTCCGCACTTGGTTGTGTAGGCTGGAGCTGCTTC |
| pgi_gluc7 | *rpoS* | CTGTTCGAGCTGAACGCCAAACAGCGTGAAGTGCTGGCACGTTGATTCGGTTTGCTGGGGTATAGGGATAACAGGGTAATATTCCGGGGATCCGTCGACC | GGCCAATTTCACGACCTACATCTTCCAGTGTTGCCGCTTCGTACCCCAGCAAACCGAATCAACGTGCCAGCACTTCACGCGTGTAGGCTGGAGCTGCTTC |
| pgi_gluc2 | *udhA* | AAAGCAAAGGCCGCTCAGGATATAGCCAGATAAATGACGGGGATCAATTGACTTACCCGCGATtagggataacagggtaatattccggggatccgtcgacc | TGTTCTTATACATAAAAGCAACAGAATGGTAACATTTTATCGCGGGTAAGTCAATTGATCCCCGTCATTTATCTGGCTATAgtgtaggctggagctgcttc |
| pgi_gluc2 | *pntA* | TTTGTGCAAGCGGGCGCTGAAATTGTAGAAGGGAATAGCGTCTGGCAGTAAGAGATCATTCTTAGGGATAACAGGGTAATATTCCGGGGATCCGTCGACC | TAACGCAATTTCATCATCTAACGGCGCATTGACCTTCAGAATGATCTCTTACTGCCAGACGCTATTCCCTTCTACAATTTGTGTAGGCTGGAGCTGCTTC |
