# Supplementary material for: Genetic Basis of Growth Adaptation of Escherichia coli after Deletion of pgi, a Major Metabolic Gene
Source: PLoS Genet. 2010 Nov 4;6(11):e1001186. doi: 10.1371/journal.pgen.1001186 (PMC2973815; doi:10.1371/journal.pgen.1001186)
Supplement: Table S3 — Primers used for Sanger sequencing to confirm mutations reported by Nimblegen and Illumina sequencing technologies. The rpoS, udhA, pntA, and pntB genes were sequenced in their entirety in all nine evolved strains using Sanger sequencing, not just regions immediately surrounding reported mutations. Primers were designed for this purpose by dividing the four genes into regions of approximately 800–900 base pairs with an overlap between regions of approximately 100 base pairs. (0.06 MB DOC) [file pgen.1001186.s003.doc]

**Table S3. Primers used for Sanger sequencing to confirm mutations reported by Nimblegen and Illumina sequencing technologies.**

| Strain | Mutation | Left primer | Right Primer |
| --- | --- | --- | --- |
| pgi_gluc1 | rpoB | GAAAGGTATCGGCGACAAGA | CCGTCGGAGTTAGCACAATC |
| pgi_gluc1 | rpoC | GTATGTGCGCACTGCTACG | AACGATGCCGCGGAGAT |
| pgi_gluc1 | rep | CACCAACCTTAGGGCTGAAA | AGCAACGTCGGCAATAAAAT |
| pgi_gluc1 | fabZ | GCTGCAACCCAACAAAGTCT | ATGGGGTCCAACGATACAAA |
| pgi_gluc3 | rpoA | GGTTATGTGCCGGCTTCTAC | GCTCTTTCGCTTTAGGCAGA |
| pgi_gluc3 | cpxR | GCATCATTTGCTCCCAAAAT | TCGAGCTTGGGTAACATCAA |
| pgi_gluc4 | cpxR | GCATCATTTGCTCCCAAAAT | TCGAGCTTGGGTAACATCAA |
| pgi_gluc4 | yfeH | ATACGCTCTCCTGCACGACT | GGTCAGTTTTCGCAATCCAT |
| pgi_gluc5 | fruK | ACATGCGCTACGTGAACAAG | CACGGGTTTCAACTTTCACC |
| pgi_gluc6 | rpoA | GGTTATGTGCCGGCTTCTAC | GCTCTTTCGCTTTAGGCAGA |
| pgi_gluc7 | rodA | GAGCGTTTACGTGACCACAA | TGCATCAGGAAGAACCACAG |
| pgi_gluc8 | cyaA | AAATGAATGGCTGGATCTCG | ACGCCACCAGTTTATTCAGG |
| pgi_gluc8 | bipA | GTCCGTTCCAGATGCAGATT | AGAACGGAAGCCAATCAGAC |
| pgi_gluc10 | rpoA | GGTTATGTGCCGGCTTCTAC | GCTCTTTCGCTTTAGGCAGA |
| pgi_gluc10 | ispU | GGAACCTGGGTAAAACAGCA | GTCTGCACCGACATAACGTG |
|  | rpoS-1 | CAGTTCAACACGCTTGCATT | TTACGATGTGAATCGGCAAA |
|  | rpoS-2 | ATACGCAACCTGGTGGATTC | CGTATGGGCGGTAATTTGAC |
|  | udhA-1 | CAATAAAACGTCAGGGCAAAA | ATAAGTACATGGCGCGGTTC |
|  | udhA-2 | CATCCACGCATTTACGACAG | GCCCACTTCATATGGCACTT |
|  | udhA-3 | CGCTGGTAAAAGGCGAAG | GCGATGGGGTTGTTTATCTG |
|  | pntA-1 | AAAAACAGCCGTATCAGCATC | GGCGGCAAAGAGTTCCATTT |
|  | pntA-2 | CGGCAAAAGTGATGGTGATT | CGGTGAGCAGGTACATTTTTC |
|  | pntA-3 | GTGCGGGCGAAATTACCT | CGGCGATACCGAAGTTGTTA |
|  | pntB-1 | CGGAGCACTGTTGCAGATT | ATTATCAGCAATGCCAGCAC |
|  | pntB-2 | AACCATTGATGCTGCCAAAC | TCGTTAGCACCAATCACCAG |
|  | pntB-3 | CGTTTGCCTGGACATATGAA | GTTGTTAGGACCGGTGGGTA |
| pgi_gluc2 | e14 deletion, region 1 | TTCCCGGAACATTGTGGTAT | GCGGGTGTAAGGAGTGGTAA |
| pgi_gluc2 | e14 deletion, region 2 | CTGGAAAAAGCCAATCCAAA | TCTCGTACAGCATGGCTCTG |
| pgi_gluc2 | e14 deletion, region 3 | GGCGTCATCATCAGTCAGAA | CAACCTACATGTGGTATATC |
| pgi_gluc2 | e14 deletion, region 4 | TTCCCGGAACATTGTGGTAT | CAACCTACATGTGGTATATC |

The *rpoS, udhA, pntA,* and *pntB* genes were sequenced in their entirety in all nine evolved strains using Sanger sequencing, not just regions immediately surrounding reported mutations. Primers were designed for this purpose by dividing the four genes into regions of approximately 800–900 base pairs with an overlap between regions of approximately 100 base pairs. Region numbers for e14 deletion confirmation refer to those shown in Figure 3.
